# Comparison of basophil activation test and lymphocyte transformation test as diagnostic assays for drug hypersensitivity

**DOI:** 10.1186/2045-7022-4-S3-P30

**Published:** 2014-07-18

**Authors:** Takeya Adachi, Hayato Takahashi, Takeru Funakoshi, Hiroyuki Hirai, Akihiko Hashiguchi, Masayuki Amagai, Keisuke Nagao

**Affiliations:** 1 Keio University School of Medicine, Department of Dermatology, Japan; 2 BML, Inc., Advanced Medicine and Development, Japan; 3 BML, Inc., Cell Biology Section, Japan

## 

Lymphocyte transformation test (LTT) is a widely used assay to detect drug-specific T cell reaction, but its relatively low sensitivity renders risk for pseudo-negativity. Basophil activation test (BAT) is an emerging assay initially used to diagnose food allergy, but its application to drug hypersensitivity as been reported anecdotally. Although a promising assay, the clinical settings or a particular class of drugs in which BAT is useful has not been well characterized. Herein, we performed a side-by-side comparison of BAT and LTT in detecting culprit drugs in several types of drug hypersensitivities. 248 patients who visited our department from October 2011 to December 2013 were enrolled in this study. Peripheral blood cells drawn from patients were co-cultured with or without relevant drugs. LTT was performed according to a standard protocol with 3H-thymidine uptake and stimulation index of 1.8 and above (CPM value with drug divided by CPM without drug) were defined positive. For BAT, whole blood cells were exposed to drugs for 24hrs and were assessed via flow cytometry for the proportion of cells that had upregulated CD203c (activated state) among total basophils. 9.0% and above were defined positive, based on values from healthy controls (mean value + 5SD). Overall, 14.7% and 28.8% of culprit drugs tested yielded positive results by BAT and LTT, respectively. Analysis of disease phenotype of patients revealed that BAT yielded positive results not only in immediate-type, but also delayed-type hypersensitivity such as drug-induced hypersensitivity syndrome and Stevens-Johnson syndrome. Of note, samples that yielded positive for LTT and BAT did not overlap, suggesting that the two analyses might compensate each other for pseudo-negativity. Furthermore, analysis focusing on the class of antibiotics showed that macrolides were frequently obtained positive via BAT, whereas that for -lactams tended to be positive via LTT. Utilizing both BAT and LTT, we screened antibiotics and NSAIDs for selection of drugs that could be used for challenge tests in patients with histories of multiple drug allergy. The negative indicative value was 96.4%, suggesting the usefulness of these assays in safely identifying drugs for future use. Taken together, different spectrum of drugs can be covered by performing both BAT and LTT, allowing identification of culprit drugs with higher sensitivity than by performing a single assay.

